# Lapachol and synthetic derivatives: *in vitro* and *in vivo* activities against *Bothrops* snake venoms

**DOI:** 10.1371/journal.pone.0211229

**Published:** 2019-01-28

**Authors:** Marcelo A. Strauch, Marcelo Amorim Tomaz, Marcos Monteiro-Machado, Bruno Lemos Cons, Fernando Chagas Patrão-Neto, Jhonatha da Mota Teixeira-Cruz, Matheus da Silva Tavares-Henriques, Pâmella Dourila Nogueira-Souza, Sara L. S. Gomes, Paulo R. R. Costa, Edgar Schaeffer, Alcides J. M. da Silva, Paulo A. Melo

**Affiliations:** 1 Laboratório de Farmacologia das Toxinas, Instituto de Ciências Biomédicas—Universidade Federal do Rio de Janeiro, Rio de Janeiro-RJ, Brazil; 2 Instituto Vital Brazil, Niterói-RJ, Brazil; 3 Laboratório de Química Bioorgânica, Instituto de Pesquisas de Produtos Naturais Walter Mors-Universidade Federal do Rio de Janeiro, Rio de Janeiro-RJ, Brazil; 4 Laboratório de Catálise Orgânica, Instituto de Pesquisas de Produtos Naturais Walter Mors-Universidade Federal do Rio de Janeiro, Rio de Janeiro-RJ, Brazil; Universidad de Costa Rica, COSTA RICA

## Abstract

**Background:**

It is known that local tissue injuries incurred by snakebites are quickly instilled causing extensive, irreversible, tissue destruction that may include loss of limb function or even amputation. Such injuries are not completely neutralized by the available antivenins, which in general are focused on halting systemic effects. Therefore it is prudent to investigate the potential antiophidic effects of natural and synthetic compounds, perhaps combining them with serum therapy, to potentially attenuate or eliminate the adverse local and systemic effects of snake venom. This study assessed a group of quinones that are widely distributed in nature and constitute an important class of natural products that exhibit a range of biological activities. Of these quinones, lapachol is one of the most important compounds, having been first isolated in 1882 from the bark of *Tabebuia avellanedae*.

**Methodology/Principal findings:**

It was investigated the ability of lapachol and some new potential active analogues based on the 2-hydroxi-naphthoquinone scaffold to antagonize important activities of *Bothrops* venoms (*Bothrops atrox* and *Bothrops jararaca*) under different experimental protocols *in vitro* and *in vivo*. The bioassays used to test the compounds were: procoagulant, phospholipase A_2_, collagenase and proteolytic activities *in vitro*, venom-induced hemorrhage, edematogenic, and myotoxic effects in mice. Proteolytic and collagenase activities of *Bothrops atrox* venom were shown to be inhibited by lapachol and its analogues 3a, 3b, 3c, 3e. The inhibition of these enzymatic activities might help to explain the effects of the analogue 3a *in vivo*, which decreased skin hemorrhage induced by *Bothrops* venom. Lapachol and the synthetic analogues 3a and 3b did not inhibit the myotoxic activity induced by *Bothrops atrox* venom. The negative protective effect of these compounds against the myotoxicity can be partially explained by their lack of ability to effectively inhibit phospholipase A_2_ venom activity. *Bothrops atrox* venom also induced edema, which was significantly reduced by the analogue 3a.

**Conclusions:**

This research using a natural quinone and some related synthetic quinone compounds has shown that they exhibit antivenom activity; especially the compound 3a. The data from 3a showed a decrease in inflammatory venom effects, presumably those that are metalloproteinase-derived. Its ability to counteract such snake venom activities contributes to the search for improving the management of venomous snakebites.

## Introduction

Snakebites occur worldwide causing disabling injuries and death with serious social impact, most often in Africa, Asia and Latin America [1,2]. This burden of human suffering caused by snakebite is ignored by most of the global health community and overlooked by development agencies and governments. Only in April 2009 this problem was included in the World Health Organization List of Neglected Tropical Diseases [3]. Annually in the world it is estimated that more than two million ophidian accidents occur, resulting in 400,000 amputations and around 125,000 deaths [4–7]. Severe venom-induced tissue damage derives from a cocktail of pharmacologically active proteins and toxins, which exhibit various enzymatic and non-enzymatic properties [7,8]. Snakes from the *Bothrops* genus are responsible for the vast majority of ophidian accidents in Central and South Americas, and *Bothrops atrox* is the major representative of this genus in the Brazilian Amazon.

The venoms of other snakes in the *Bothrops* genus also have notable biological actions, however, with varying intensities. As it relates to hemorrhagic activity, *Bothrops jararaca* is known to have one of the most intense actions. Because of this there are numerous studies involving *B*. *jararaca* venom and its hemorrhagic effects, such as the isolation and characterization of jararhagin, the first metalloproteinase isolated from *B*. *jararaca* venom with its complete primary structure characterized [9]. This study opened new doors for protein classification and structure/function studies of snake venom metalloproteinases (SVMP’s).

**Fig 1:**
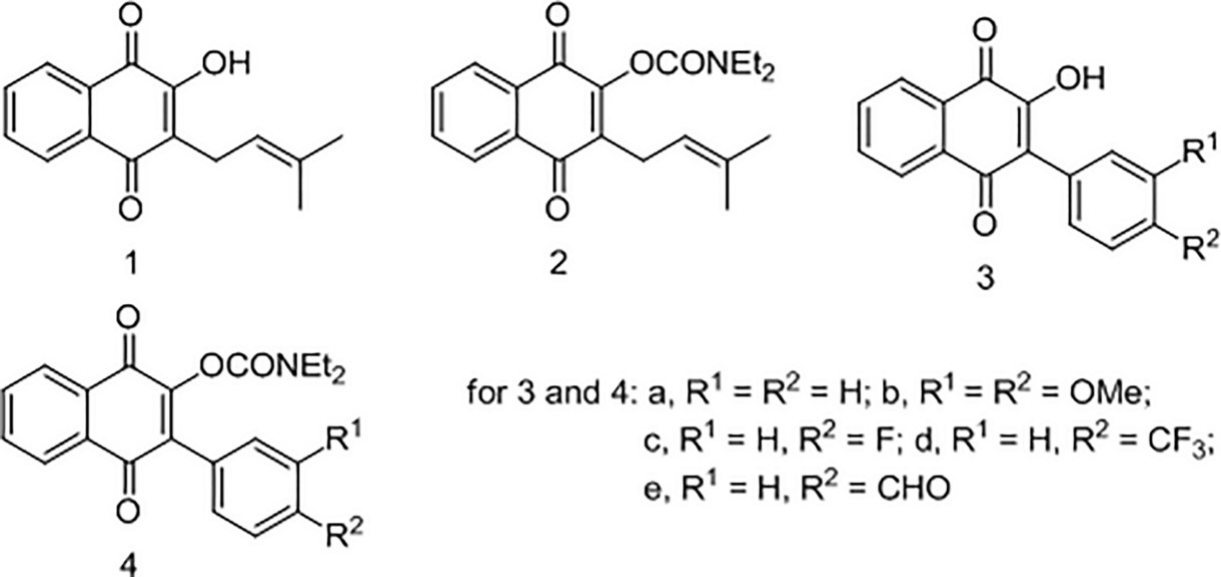
Lapachol (1) and analogues (2–4).

The recommended treatment for ophidian accidents is either specific or polyvalent antivenoms, which in turn have limited effectiveness against the toxins involved in local tissue effects [10–14]. The use of potentially medicinal plants to halt the effect of snake venoms has been proposed by previous studies [15–19]. Our group has been particularly interested in the search for new and effective pharmacologically active plant compounds used in folk medicine to treat or prevent damage caused by accidents with venomous snakes [20–24]. *Tabebuia impetiginosa* (Bignoniaceae) is an evergreen, canopy tree, with pink, yellow, white and purple flowers found predominantly in the Amazon rain forest, but can also be found all over South America [25]. Lapachol (2-hydroxy-3-(3-methyl-2-butenyl-)-1,4-naphtoquinone, C_15_H_14_O_3_, molecular weight 242.2738 g/mol. (Fig 1) was first isolated from *Tabebuia impetiginosa* in 1858, and was initially shown to exhibit antimalarial, antitumor, antifungal, leishmanicidal, bactericidal and antiparasitic activities. Due to its toxicity, however, studies were eventually discontinued [26–27]. Lapachol has been used as starting point to obtain new bioactive quinones that exhibit interesting pharmacological profiles [28–30]. Previous work with naphthoquinones structurally related to lapachol demonstrated activity against muscle damage induced by *Bothrops jararacussu* venom [29]. The present research was aimed at exploring, for the first time, the activity of lapachol against snake venoms. This prompted us to study the potential activity of the analogues based on the 2-hydroxi-naphthoquinone scaffold, which had been previously synthesized (Fig 1) [31], investigating their effects against some important activities of crude *Bothrops atrox* and *Bothrops jararaca* venoms under different experimental protocols *in vivo* and *in vitro*.

## Material and methods

*Bothrops jararaca* and *Bothrops atrox* venoms were collected from 15–30 snakes above 4 years old belonging to the serpentarium of Instituto Vital Brazil, Niterói, Brazil; creatine kinase (CK) activity was determined using a CK NAC kit from BIOCLIN; adult male Swiss mice were provided by the Rodent Vivarium of the Institute of Microbiology Paulo de Góes–UFRJ (Federal University of Rio de Janeiro). Mice weighing 25.0 ± 1.0 g used for the study received water and food *ad libitum* and were kept under a natural light cycle. Lapachol was purchased from Sigma Aldrich, St.Louis, USA, and lapachol analogues were obtained by the Institute for Natural Products Research (Instituto de Pesquisas de Produtos Naturais Walter Mors–UFRJ). All compounds were used for assessing enzymatic activities (proteolytic, phospholipase and collagenase), and of the two most efficient ones, i.e. compounds 3a and 3b, the first was chosen to be tested in all *in vivo* and *in vitro* experiments due to higher availability. All protocols were approved by the Ethics Committee for the Use of Animals of the Federal University of Rio de Janeiro (CEUA-UFRJ-N° DFBCICB072-04/16, following the Guide for the Care and Use of Laboratory Animals, from the National Academy of Sciences, 2011), where all the procedures that could cause pain were performed under anesthesia.

### Venom proteolytic activity

The proteolytic activity assay was carried out as previously described [32]. *Bothrops atrox* venom (10 μg/mL) was preincubated with lapachol and analogues (3–100 μM) for 30 min at 37°C. Substances were dissolved in DMSO and added up to 3 μL to solutions. Venom alone or the preincubated mixtures were added to a solution containing 400 μL of 0.2% azocasein, 200 μL of 0.2 M Tris-HCl (pH 8.8) with CaCl_2_ (20 mM) and distilled water (200 μL qsp). The reaction continued for 90 min at 37°C and was stopped by the addition of 400 μL of 15% trichloroacetic acid solution, and then centrifuged at 10,000 rpm. Then 1.0 mL of the supernatant was collected and mixed with 0.5 mL NaOH (2.0 M). This final solution was analyzed by spectrophotometry at an absorbance of 420 nm. Negative controls with pure DMSO were used for each compound and its absorbance subtracted from treatment group.

### Venom collagenase activity

*Bothrops atrox* venom collagenase activity was assessed by a colorimetric method adapted from previous studies [33]. A 0.3% azo-dye impregnated collagen solution (Azocoll, Sigma Aldrich, St.Louis, USA) was prepared with Tris-HCl 0.2 M pH 7.5 as buffer. To each assay tube 200 μL of this solution was added, plus 6 μL of a solution of 1 M CaCl_2_ (20 mM) and final volume (300 μL) adjusted with distilled water. Venom (50 μg/mL), lapachol and analogues at different concentrations (10–100 μM) were added and its volumes were subtracted from distilled water. Tested substances were dissolved in DMSO and added up to 10 μL to solutions. Tubes were maintained at 37°C for 90 min and stirred gently every 10 min. At the end of this period, tubes were centrifuged at 10,000 rpm for 2 min and the supernatant absorbance was measured on spectrophotometry at an absorbance of 520 nm. Negative controls with pure DMSO were used for each compound and its absorbance subtracted from treatment group.

### Hemorrhagic activity

Besides *B*. *atrox*, the venom of *B*. *jararaca* was also used in the protocol of hemorrhagic activity due to its marked ability to cause bleeding. The hemorrhagic effect was induced by an intradermic (i.d.) injection of 0.1 mL of *B*. *jararaca* and *B*. *atrox* venom (1 mg/kg) in the abdomen of mice and quantified as previously described [21]. The negative control received 0.1 mL of physiological saline solution (PSS) (mM: 135 NaCl, 5 KCl, 2 CaCl_2_, 1; MgCl_2_, 1 NaHPO_4_, 15 NaHCO_3_, 11 dextrose) i.d. injection. To evaluate the antihemorrhagic activity of 3a compound, *Bothrops* venom dissolved in PSS was first incubated with the 3a (1–10 mg/kg) for 15 min at room temperature prior to i.d. injection. Two hours after the venom injection, animals were killed under anesthesia, and the skin covering the abdomen was removed, stretched, and dried at room temperature for 72 h. The skin was then fixed to a lucite base plate, and the entire area at the injection site and the surrounding area were transilluminated using an incandescent light. Light transmitted over an area of 109 mm^2^ was read and registered as arbitrary units of absorbance (a.u.).

### Thigh edema

The induction of edema was evaluated by an intramuscular injection of 50 μL *Bothrops atrox* venom (1 mg/kg) at the posterior aspect of the right thigh, and PSS was used as negative control. To evaluate the anti edematogenic activity, the venom dissolved in PSS was first incubated with lapachol and analogues 3a and 3b (3–30 mg/kg) for 15 min at room temperature prior to injection. Edema was measured with a digital micrometer caliper, evaluating the anteroposterior length and the mediolateral width of mice thigh at 0, 15, 30, 60, 90, 120 min [34].

### Phospholipase A_2_ activity

Phospholipase A_2_ activity was assessed by adapting the turbidimetric assay described previously [35]. Substrate was prepared with a 10% chicken egg yolk solution in 150 mM NaCl. Each assay tube was prepared by taking a final volume (0.25 mL) of a 0.6% dilution of the egg suspension and adding it to a solution containing 150 mM NaCl, 10 mM CaCl_2_, 0.01% taurocholic acid, and 5.0 mM Tris–HCl (pH 7.4). The reactions were started by adding 10 μg/mL of *Bothrops atrox* venom alone or preincubated for 30 min at 37°C with lapachol and analogues (100 μM). The absorbance of the solutions was read in ELISA at 925 nm before and 30 min after starting the reactions, and data was expressed as percentage of venom activity [34].

### Myotoxicity *in vitro*

Muscle damage *in vitro* was assessed by measuring the rate of creatine kinase (CK) release from isolated mouse muscles and performed as previously described [21, 36, 37]. Briefly, mouse extensor digitorum longus (EDL) muscle was removed and superfused continuously with PSS. The pH of this solution was equilibrated to 7.3 with carbogen. During the superfusion, the muscles were exposed to *Bothrops atrox* venom (25 mg/mL), and venom plus analogue 3a (100 μM) that was added to PSS. Perfusion samples were collected at 30 min intervals and replaced with fresh solution. The collected samples were stored at 4°C and their CK activity was determined according to previously described procedures [36, 38]. At the end, muscles were weighted. The CK activity was expressed in international units (U), where 1 U is the amount that catalyzes the transformation of 1 μmol of substrate at 25°C. The rate of CK release from the isolated muscle was expressed as enzyme units released into the medium per gram per hour of collection (U.g^-1^. h^-1^).

### Myotoxicity *in vivo*

Myotoxicity of *B*. *atrox* venom was assessed *in vivo* by measuring the increase of plasma CK activity induced by intra-muscular (i.m.) injection of venom alone or associated with lapachol or analogues 3a and 3b. The venom was dissolved in PSS to a final volume of 0.1 mL (1.0 mg/kg) and was injected into the rear thigh of the mice as described previously [21, 37, 38]. Negative controls consisted of mice injected with the same volume of PSS. To evaluate the antimyotoxic activity, the venom dissolved in PSS was first incubated with lapachol and analogues 3a and 3b (30 mg/kg) for 15 min at room temperature prior to injection. Blood was collected under anesthesia from the orbital plexus with heparinized capillaries, immediately before and two hours after venom injection, and CK activity in plasma was determined with UV spectrophotometry at 340 nm, according to previously described procedures [36,38].

### Clotting time

The clotting time was assessed by the modified Lee-White method [39]. The animals were grouped and about 50 μL of blood were collected from the orbital plexus using non-heparinized microhematocrit capillary tubes. Before collecting blood, the tubes were filled with 20 μL of PSS; *Bothrops atrox* crude venom (1 μg/μL); and venom pre-incubated for 30 min with 3a compound (100 μM). The clotting time was determined and compared among the different groups (three groups with 8–11 mice each).

### Statistical analysis

Data were expressed as mean ± SEM. The number of experiments performed is provided in the legends of the figures, for instance as (n = 5), meaning there were five samples analyzed in one experiment. One-Way Analysis of Variance (ANOVA) was used to compare groups with one variable, followed by Dunnett's post-hoc test. For two variables, the Two-Way Analysis of Variance (ANOVA) was used followed by Bonferroni's post-hoc test. The p value <0.05 was used to indicate a significant difference between means. The software GrapPad Prism version 5.01 was used to provide statistical analysis. Graphs were made using Sigmaplot program version 10.0.

## Results

### Proteolytic activity

**Fig 2:**
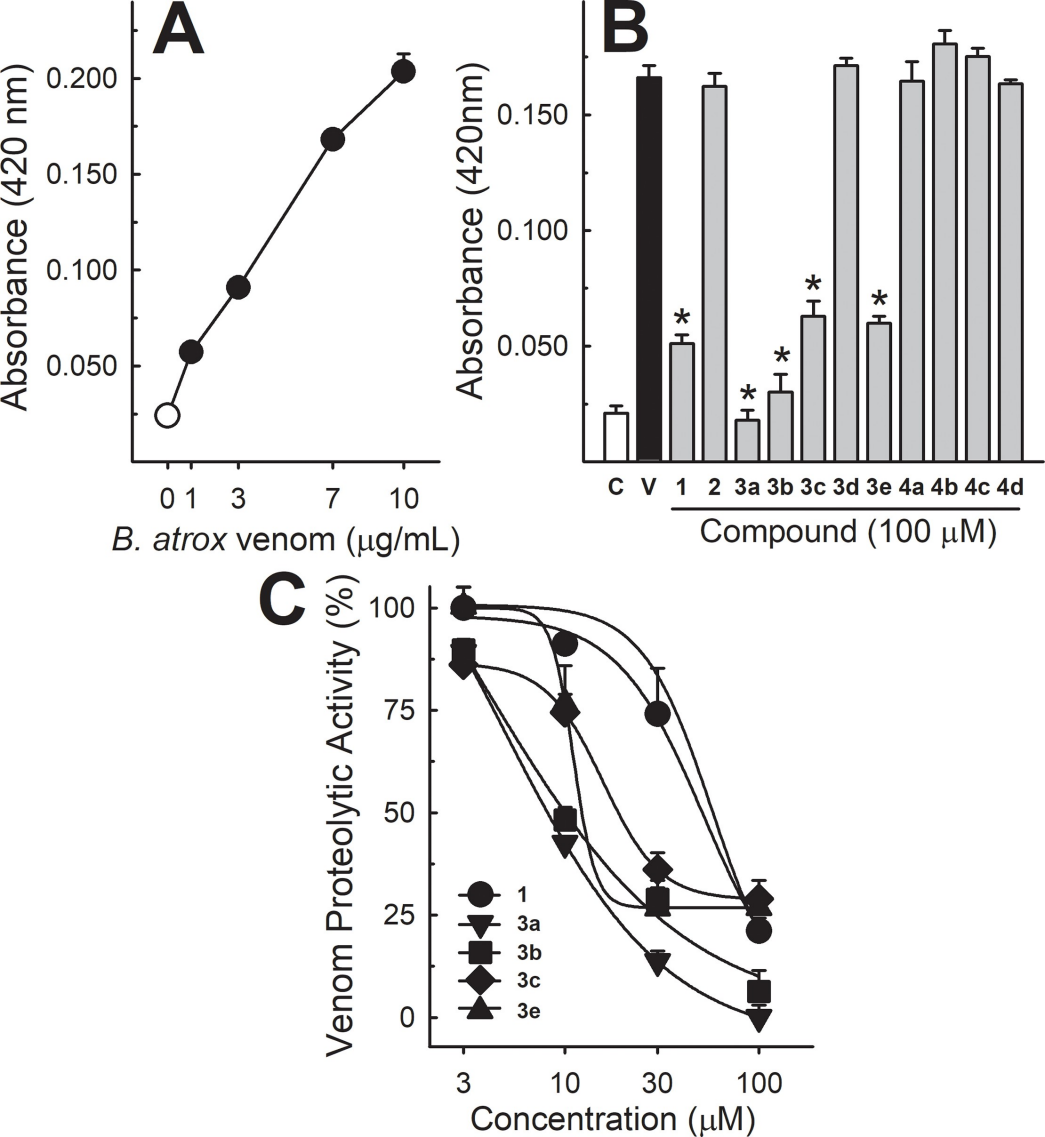
Proteolytic activity of *B*. *atrox* venom, and the effect of lapachol and analogues. Panel A shows proteolytic activity curve of *B*. *atrox* venom (1–10 μg/mL) (n = 5). Panel B shows the inhibition of *B*. *atrox* venom (10 μg/mL) by lapachol (1) and analogues (numbers 2–4) at 100 μM (n = 5). Panel C shows the inhibition of proteolytic activity by lapachol and compounds 3a, 3b, 3c and 3e in a concentration-dependent way (n = 5). One-way ANOVA Dunnett’s post-hoc test * p<0.05 *vs B*. *atrox* venom (Panel B).

*Bothrops atrox* venom induced the hydrolysis of azocasein in a concentration-dependent manner (Fig 2, panel A). The incubation of 10 μg/mL of the venom with lapachol and its analogues (100 μM, Fig 2, panel B) antagonized its proteolytic activity, being inhibited in a concentration-dependent manner by lapachol (1) and the analogues 3a, 3b, 3c, 3e (3–100 μM) (Fig 2, panel C). Analogues 2 and 4 showed no effect.

### Collagenase activity

**Fig 3:**
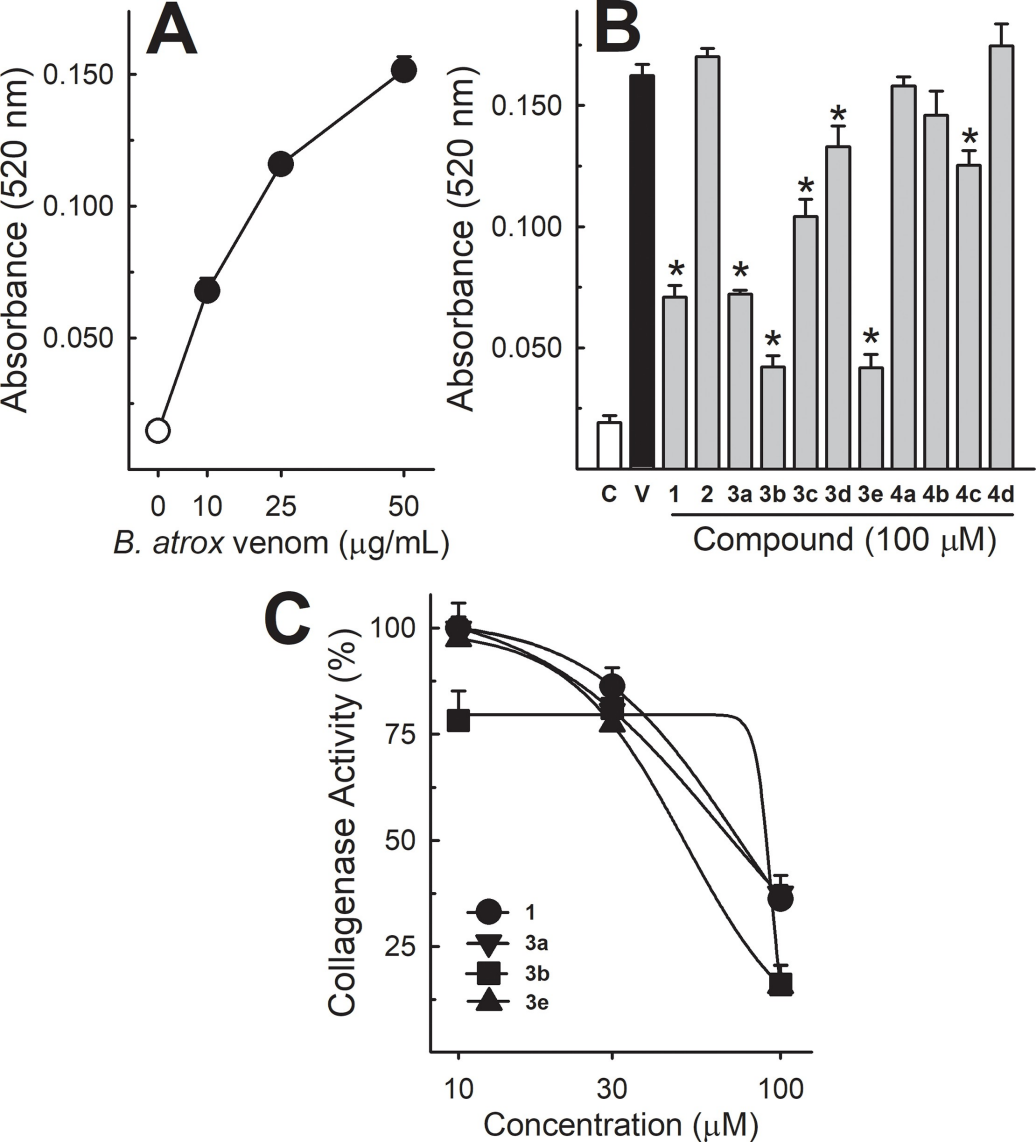
Collagenase activity of *B*. *atrox* venom, and the effect of lapachol and analogues. Panel A shows the collagenase activity curve of *B*. *atrox* venom (10–50 μg/mL) (n = 5). Panel B shows the inhibition of *B*. *atrox* venom (50 μg/mL) by lapachol (1) and analogues (numbers 2–4) at 100 μM (n = 5). Panel C shows the inhibition of collagenase activity by compounds 1, 3a, 3b and 3e in a concentration-dependent way (n = 5). One-way ANOVA Dunnett’s post-hoc test * p<0.05 *vs B*. *atrox* venom.

*Bothrops atrox* venom induced the release of the Azo group from the substrate (Azocoll), increasing the absorbance in a concentration-dependent manner (Fig 3, panel A). The incubation of 50 μg/mL of the venom with lapachol and analogues 3a, 3b and 3e (100 μM, Fig 3, panel B) antagonized its collagenase activity. Analogues 2 and 4 showed no effect. Lapachol (1) and analogues 3a, 3b, 3e (10–100 μM, Fig 3, panel C) antagonized venom collagenase activity in a concentration-dependent manner.

### Hemorrhagic activity

**Fig 4:**
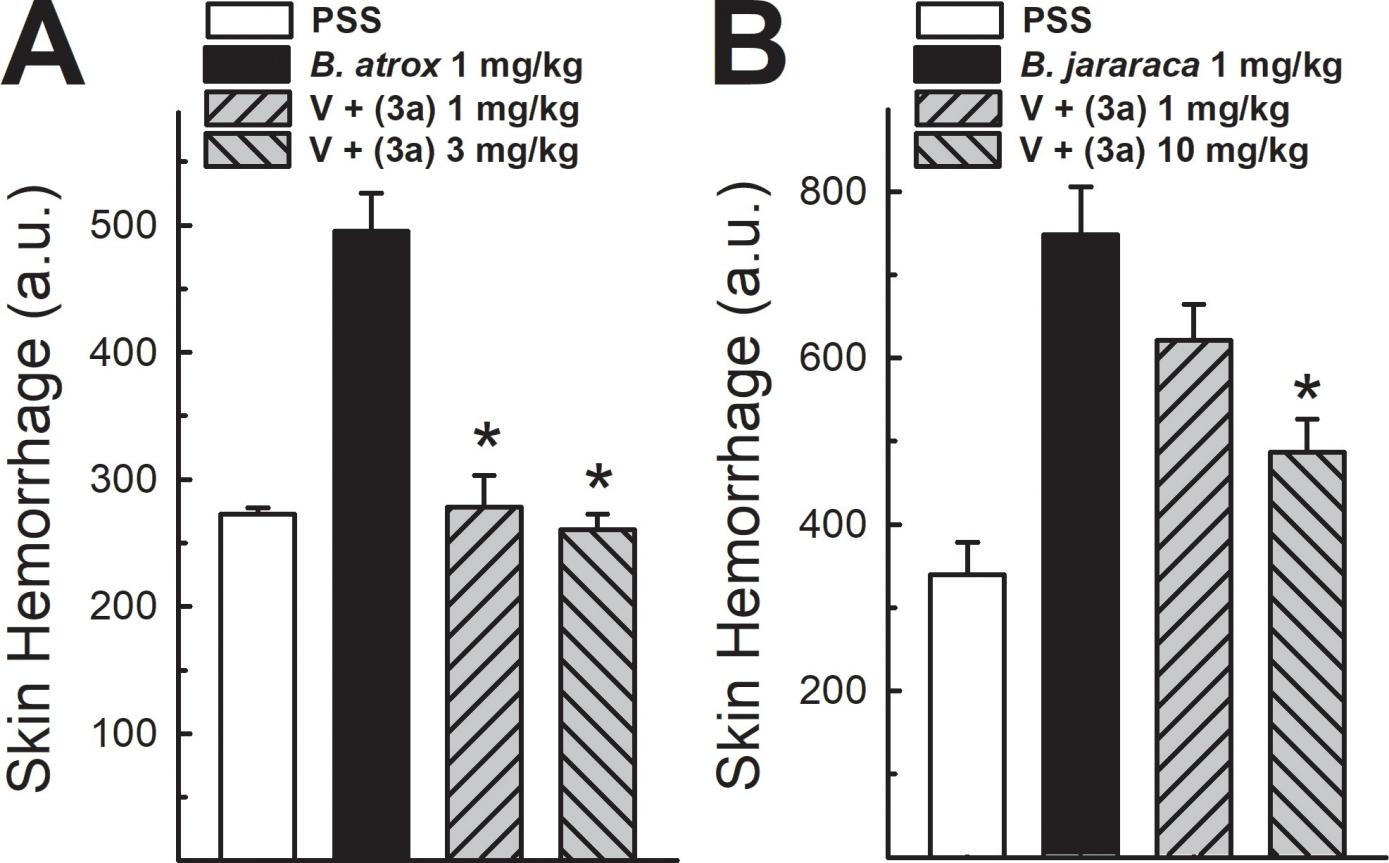
Hemorrhagic activity of *Bothrops* venoms in mouse skin: Effect of analogue 3a. Data demonstrates skin hemorrhage after intradermic injection of *Bothrops* venom in the presence of analogue 3a at 1 and 3 mg/kg (for *B*. *atrox*, panel A) and 1 and 10 mg/kg (for *B*. *jararaca*, panel B) (n = 8). One-way ANOVA Dunnett’s post-hoc test * p<0.05 *vs B*. *atrox* venom.

The intradermic injection of *Bothrops* venoms (1 mg/kg) in mice induced a severe hemorrhagic skin damage surrounding the region injected, with measurement of light absorbance and expression in arbitrary units (a.u.). The value of absorbance was around 250–350 a.u. with PSS injection, while venom injection reached up to 500 a.u. for *B*. *atrox* and 760 a.u for *B*. *jararaca*, respectively. The venom preincubation with compound 3a at 1 mg/kg and 3 mg/kg abolished hemorrhage induced by *B*. *atrox* venom (Fig 4, panel A). On its turns, the inhibition of *B*. *jararaca* venom was was higher than 70% with 10 mg/kg of compound 3a (Fig 4, panel B).

### Edematogenic activity

**Fig 5:**
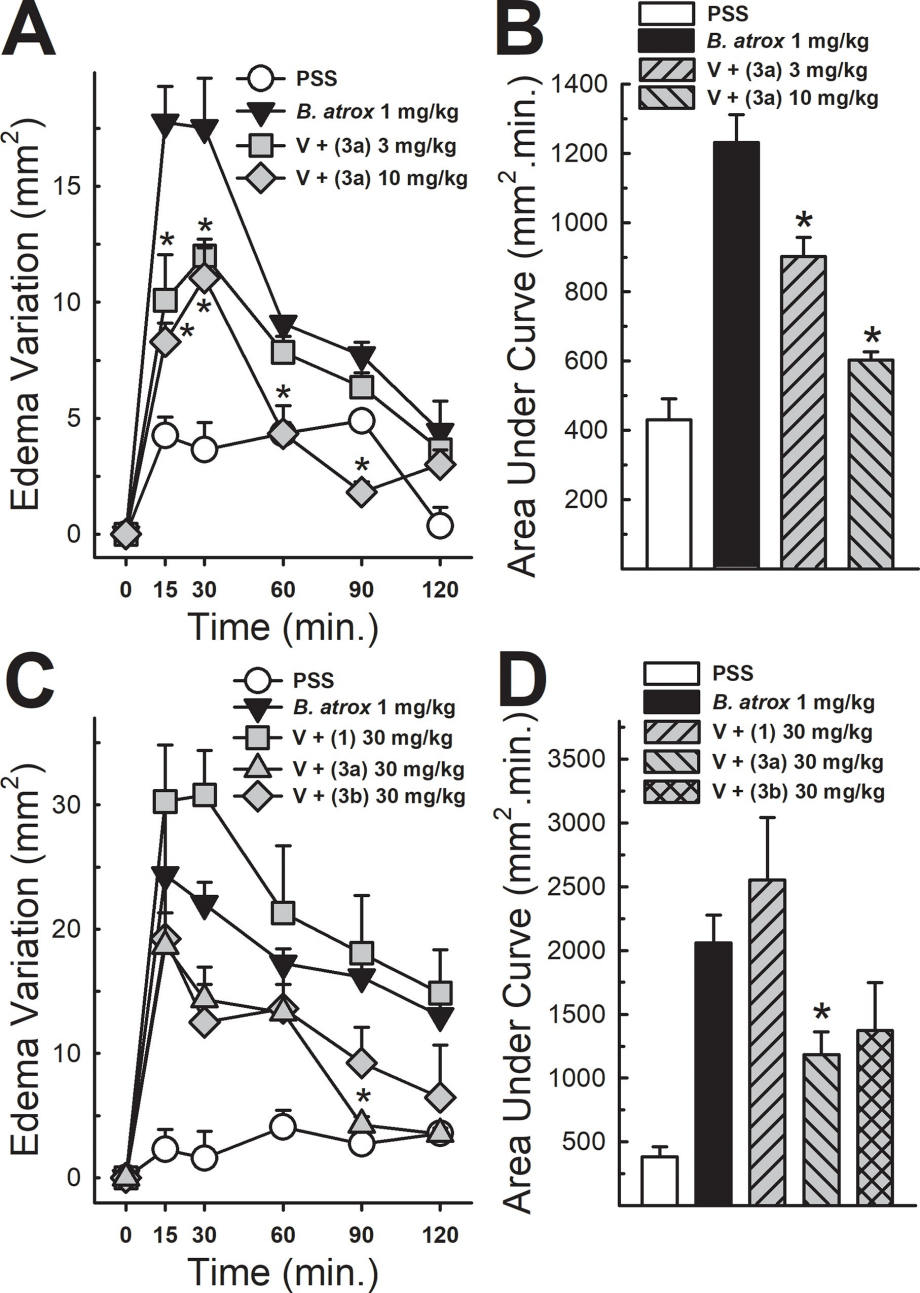
Effect of analogue 3a on *B*. *atrox* venom edematogenic activity. Panels A and B show edema variation after injection of PSS, *B*. *atrox* venom (1 mg/kg) alone or associated with analogue 3a (3 and 10 mg/kg), and resulting areas under the curves, respectively (n = 5). Panel C shows edema when incubated with lapachol (1) and analogues 3a and 3b at 30 mg/kg, with the respective areas under the curves (Panel D). (n = 5). Two-Way ANOVA Bonferroni’s post-hoc test * p<0.05 *vs B*. *atrox* venom (Panels A and C). One-way ANOVA Dunnett’s post-hoc test * p<0.05 *vs B*. *atrox* venom (Panels B and D).

Following intramuscular injection of *B*. *atrox* venom (1 mg/kg; 50 μL) into the hind limb, the animals presented a significant increase in thigh area, as represented by the area under the curve (a.u.c.) comparing with PSS injection. When preincubated with compound 3a (3 and 10 mg/kg), edematogenic activity of the venom was significantly decreased (Fig 5, panels A and B). In another set of experiments, a higher dose of compound 3a was tested along with lapachol and compound 3b, all at 30 mg/kg. Again, compound 3a significantly antagonized edematogenic activity of the venom, while compound 3b did not show statistically significant effect, nor did lapachol (Fig 5, panels C and D).

### Phospholipase A_2_ activity

**Fig 6:**
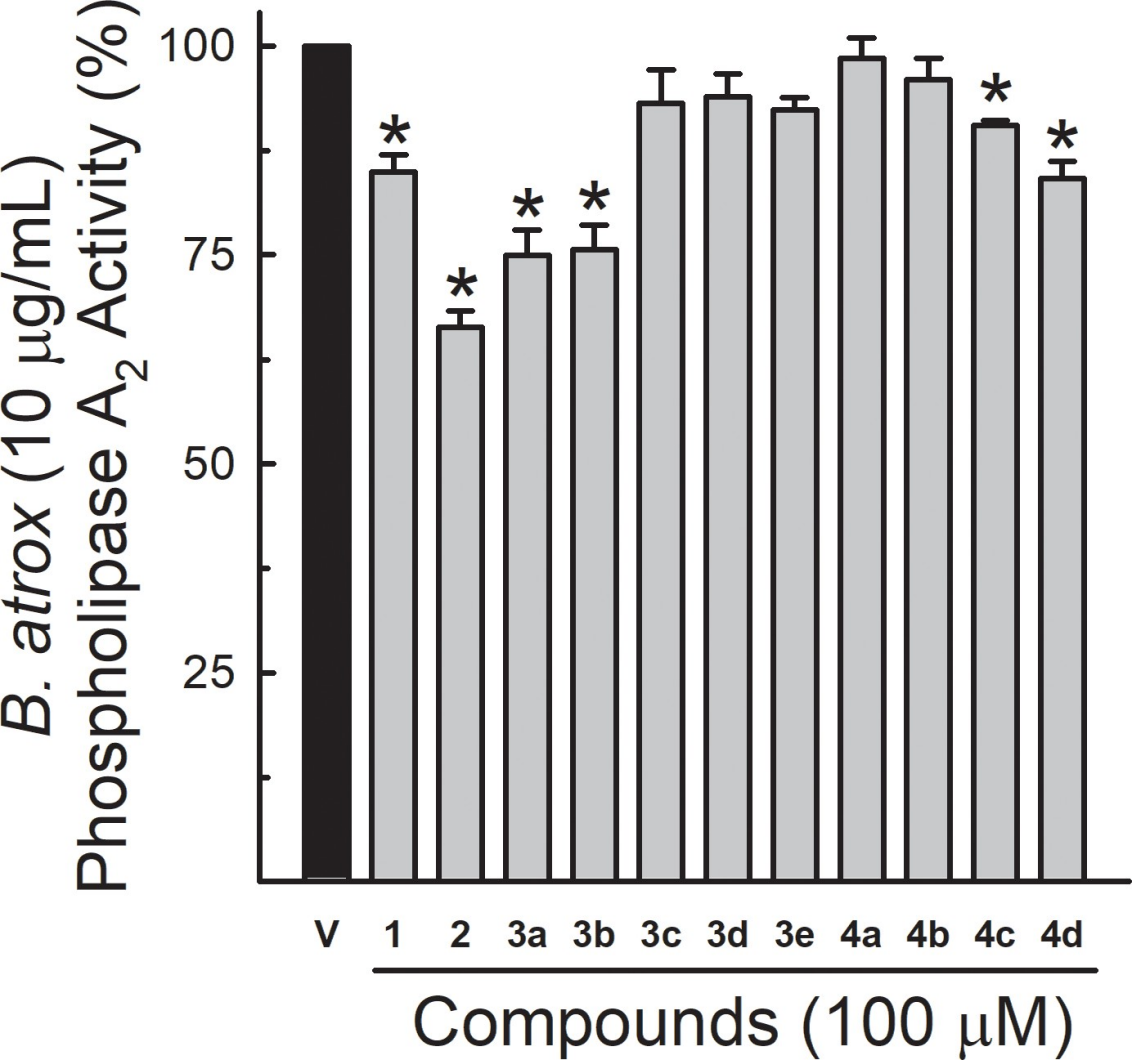
Effect of lapachol and analogues on *B*. *atrox* venom phospholipase A_2_ activity. Data show the PLA_2_ activity of *B*. *atrox* venom alone (10 μg/mL) (considered as 100% activity) or preincubated with lapachol (1) and analogues (numbers 2–4) at 100 μM (n = 6). One-way ANOVA Dunnett’s post-hoc test *p < 0.05 *vs B*. *atrox* venom.

The turbidimetric assay for phospholipase A_2_ (PLA_2_) activity showed that the venom of *B*. *atrox* reduced the turbidity of egg yolk solutions (0.360 ± 0.004 absorbance units at 925 nm, for the concentration of 10 μg/mL), compared to the negative control (0.641 ± 0.028 absorbance units). This difference was considered as 100% of venom PLA_2_ activity. Lapachol and analogues 2, 3a, 3b, 4c and 4d (100 μM) decreased the enzymatic activity of 10 μg/mL of venom down to 84.9 ± 2.0%, 66.3 ± 2.0%, 74.9 ± 3.0%, 75.6 ± 2.9%, 90.5 ± 0.6% and 84.1 ± 2.1%, respectively (Fig 6).

### Myotoxic activity

**Fig 7:**
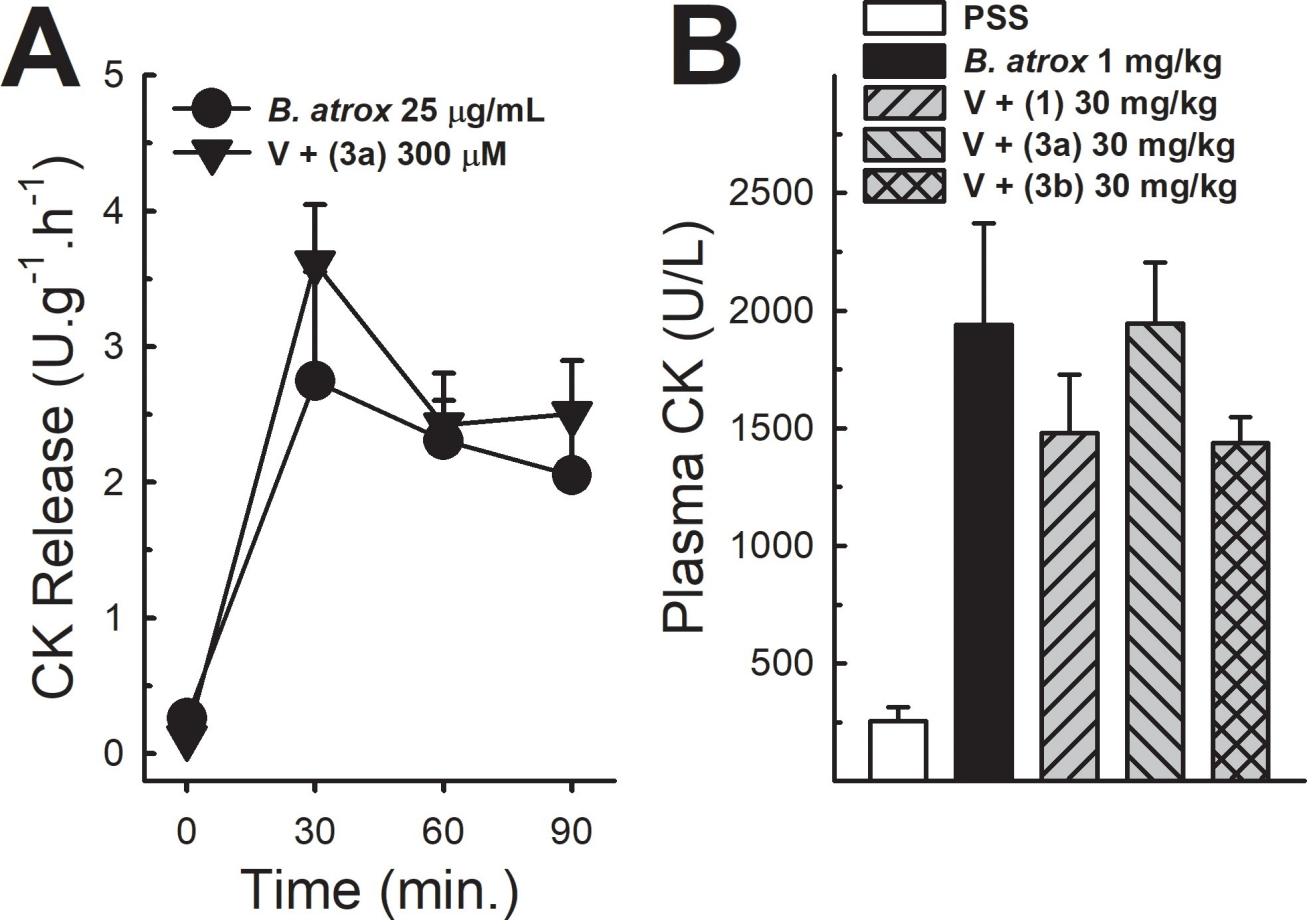
Effect of analogue 3a on *B*. *atrox* venom myotoxicity *in vitro*, and lapachol and its analogues 3a and 3b *in vivo*. Panel A shows EDL muscle superfused with *B*. *atrox* venom (25 μ/mL) alone or associated with analogue 3a (300 μM) (n = 4). Two-Way ANOVA Bonferroni’s post-hoc test. Panel B shows plasma CK 2 h after i.m. injection (0.1 mL) of *B*. *atrox* venom (V) alone or associated with lapachol (1), analogue 3a or 3b (30 mg/kg) (n = 5). One-way ANOVA Dunnett’s post-hoc test.

*In vitro* myotoxic observations demonstrated that *B*. *atrox* venom induced a time-dependent increase in the rate of CK release from basal to values that reached up to 15–20 U.g^-1^.h^-1^ after 90 min of EDL muscle exposure to venom. When we added the compound 3a to the venom solution, it did not change the rate of CK release induced by the venom (Fig 7, panel A). Besides, mice injected with the venom of *B*. *atrox* (1 mg/kg) presented, two hours after venom injection, an increased activity of CK in plasma, which ranged from 255.89 ± 59 U/L in the group receiving the PSS solution, up to 1938.63 ± 252.56 U/L in the group receiving the venom. Preincubation of venom with lapachol (1) and analogues 3a, 3b (30 mg/kg) did not significantly inhibit the myotoxic activity of the venom 2 h after venom injection (Fig 7, panel B).

### Blood clotting activity

**Fig 8:**
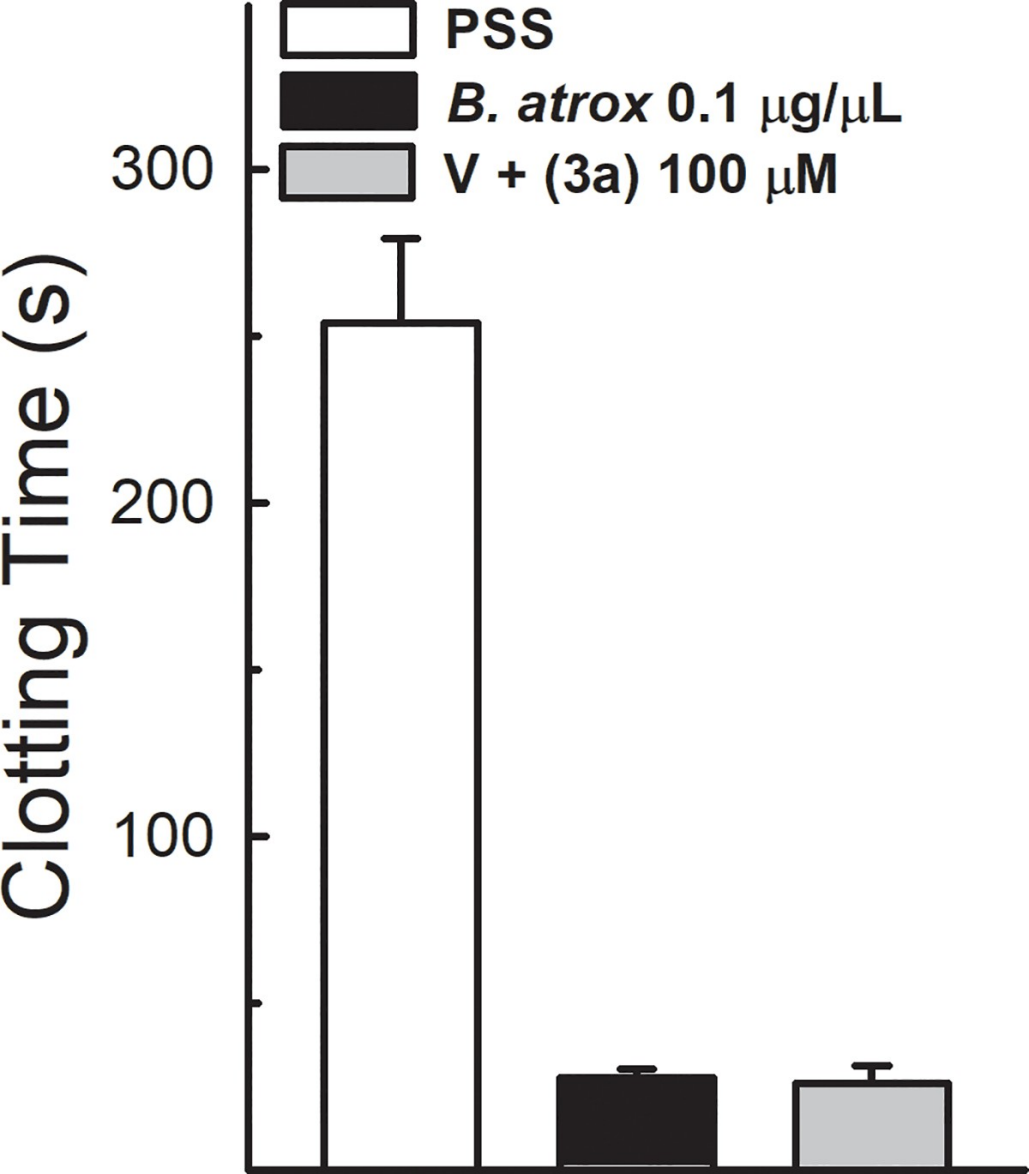
Effect of analogue 3a on *B*. *atrox* venom procoagulant activity and clotting time. Data show the time spent for mice blood collected in a capillary previously containing venom alone or associated with analogue 3a to clot (n = 8–11). One-way ANOVA Dunnett’s post-hoc test.

The venom of *B*. *atrox* (1 μg/mL) induced an intense decrease in the clotting time of 50 μL of blood collected from the conjunctival sac of anesthetized mice, from control values of 254 ± 26.05 s down to 27.28 ± 5.73 s (Fig 8). The compound 3a did not inhibit the procoagulant activity induced by the tested venom.

## Discussion

This study demonstrated that some synthetic naphthoquinones structurally related to lapachol were able to antagonize some activities of *Bothrops* snake venoms. Naphthoquinones are compounds widespread in nature playing important physiological roles in animals and plants. Previous studies described that secondary metabolites bearing in their structure the 1,4- and 1,2-naphthoquinone moieties have been isolated from plants and exhibit interesting biological activities [40]. In addition, lapachol (2-hydroxy-3-prenyl-1,4-naphthoquinone), found in *Tabebuia* species, showed to be active against the Walker-256 carcinoma and Yoshida Sarcoma cells [29, 41, 42].

The present data from experiments using *Bothrops atrox* venom as source of different active enzymes confirmed an important proteolytic activity of this crotalid venom. The proteolytic effects on the azocasein and azocollagen substrates were concentration-dependent, and those activities were significantly inhibited by lapachol and some of its synthetic analogs. These results corroborate previous investigations using compounds isolated from so-called antiophydic plants, belonging to different classes of natural products and showing antagonism of snake venom proteolytic activities [20, 24, 43, 44].

Tissue reactions and effects from *Bothrops* snakebites, such as edema, hemorrhage and necrosis, are directly correlated with toxins' enzymatic actions, mainly the proteolytic activity [7, 45–47]. For example, it is proposed that one of their targets is the basal lamina, on the structural proteins from the vascular wall and endothelial cells, such as the integrins, causing vessel disruption and hemorrhage. These proteolytic enzymes are altogether named snake venom metalloproteases (SVMPs) [46, 47]. The presence of SVMPs is well documented in *Bothrops* venoms, e.g. by hydrolysis of casein or collagen *in vitro*, and the results found in the present study are in agreement with the hemorrhagic effect observed in the mouse skin *in vivo*. Furthermore, as collagenases, they act by inducing basal lamina disruption, leading to angiorrhexis, blood extravasation and hemorrhage [45, 46, 48–51].

The results just presented showed that the proteolytic and the collagenase activities were antagonized by the lapachol analog 3a *in vitro*, as well as the skin hemorrhage induced by both *B*. *jararaca* and *B*. *atrox* venoms. We ascribe the protective effect of lapachol analogue 3a in the skin to inhibition of proteolytic and collagenase activities. Thus, this compound may have prevented the vessel degradation by protecting the basal lamina from the venom. It is relevant to emphasize the inhibition of skin hemorrhage, because this effect is considered a frequent and serious manifestation of viperidae snakebite envenoming [7, 51,52].

As well as proteolytic toxins, phospholipase A_2_ (PLA_2_) toxins are present in the snake venom composition, and when injected in the prey, usually in large muscles or subcutaneously, they cause a range of pathological effects including edema and myonecrosis. Some PLA_2_ toxins, mainly the Lys-49, named cationic myotoxins, act specifically disrupting the sarcolemma on skeletal muscles causing extensive damage [7, 53, 54]. Besides, edema is a major effect of *Bothrops* venoms, which may be explained by the inflammatory process initiated by toxins with PLA_2_ activity, which catalyzes the hydrolysis of cellular phospholipids to generate arachidonic acid and lysophospholipid, unleashing the formation of pro-inflammatory eicosanoids [23, 55–57]. Along with tissue damage, edema can be severe enough to cause functional loss or even compartmental syndrome [7, 58]. Therefore both myotoxicity and edema formation are largely related to PLA_2_ activity. The current study showed that compound 3a partially antagonized the *B*. *atrox* PLA_2_ and edematogenic activities, but not myotoxicity in mice. A possible explanation is that this partial antiedematogenic effect could be related to its anti-proteolytic activity, somewhat protecting the vessels basal lamina, while being only slightly due to its weak anti-PLA_2_ activity, which, in turn, presumably explains the lack of anti-myotoxic activity. This phenomenon helps to explain the complexity of pharmacological effects of snake venoms. Probably the best medicine against snakebites will have to likewise be a pool of substances, besides the antivenom currently used.

In summary, vascular dysfunctions leading to edema and hemorrhage is thought to be presumably metalloproteinase-derived, just like collagenases from bacteria, for instance [59, 60]. In addition, metalloprotease damage is also due to their pro-inflammatory action and to local ischemia arising from reduction of blood supply following the damage of the microvasculature [46, 61]. Different from its synthetic analog 3a, the natural compound lapachol did not reduce the edema induced by *B*. *atrox* venom, although it presents some anti-inflammatory properties, which has been shown in carrageenan inflammatory model [62]. Regalado and coworkers (2015) [63] have demonstrated an anti-inflammatory activity of the methanol extract of the stems of *Tabebuia hypoleuca* in carrageenan-induced mice edema, which contains lapachol. In the present experiments lapachol and compound 3a had a slight effect on *B*. *atrox* PLA_2_ activity, as other analogues compounds. The carrageenan inflammatory model demonstrates that inflammation involves local autacoid effects, nitric oxide mediation, ciclooxigenase activation, inflammatory cell and cytokines, and can be prevented by COX inhibitors [64–66]. Overall, the current data suggest that the aforementioned naphthoquinones, structurally related to lapachol, act mainly as potential anti-proteolytic agents.

It became clear that the aryl substitution (compound 3) improved the ability of lapachol in inhibiting enzymatic activities. From these results it was shown that the best substrate was the 2-hydroxy-3-arylnaphthalene-1,4-dione with no substitution pattern (3a), once it also antagonized edema formation and skin hemorrhage. Otherwise, the substitution of hydroxyl group in naphtoquinone by OCONEt2 (analogues 2 and 4a-4d) abolish their antienzymatic activities.

Together, the present results are in agreement with ethnobotanical studies showing that *Tabebuia aurea*, which contain lapachol, has been used as anti-inflammatory and antiophydic tool in folk medicine [67, 68]. The lapachol analogues, mainly the 3a, could help in the development of a drug with potential therapeutic applications, although further studies are needed, particularly with isolated toxins from the crude venoms which will allow for a better understanding and subsequent application of such compounds. Another feasible application of these compounds with anti-enzymatic activity is that they could help with anticancer drug research. It is noteworthy that some studies reported that a large number of metalloproteinases from cancer cells are involved in the degradation and remodeling process of the extracellular matrix, leading to tumor expansion. Many inhibitors are being designed and clinically tested [69–71]. This investigation using the snake venom as a model of enzymatic approach, either *in vitro* or *in vivo*, provides an opportunity to show that they are invaluable research tools, allowing for the testing of natural or synthetic compounds with promising enzymatic inhibition properties.

## Conclusion

In summary, some newly synthesized lapachol analogues exhibit a range of significant inhibition of enzymatic activities, suggesting potential therapeutic value against the local effects of crotalid venoms.

## Supporting information

S1 Dataset Individual results and statistical analysis for Figs 2–8. (XLSX)
